# Nurse-Led Mobile Clinics to Improve Rural Health Access and Disaster Preparedness: A Mixed-Methods Evaluation of a Texas Program

**DOI:** 10.3390/ijerph23060702

**Published:** 2026-05-26

**Authors:** Nicole Peters Kroll, Sharon L. Dormire, Kelly L. Wilson

**Affiliations:** College of Nursing, Texas A&M University, 8447 John Sharp Parkway, Bryan, TX 77807, USA; sdormire@tamu.edu (S.L.D.); kwilson@tamu.edu (K.L.W.)

**Keywords:** rural health, mobile clinics, nurse-led care, disaster preparedness, interprofessional education, telehealth, health equity

## Abstract

**Highlights:**

**Public health relevance—How does this work relate to a public health issue?**

- Nurse-led mobile clinics improve access to primary and preventive care in rural and underserved communities affected by provider shortages, transportation barriers, and geographic isolation.
- Integration of telehealth and mobile care delivery may support continuity of care and strengthen healthcare access during disasters and other public health disruptions.

**Public health significance—Why is this work of significance to public health?**

- Findings support nurse-led mobile clinics as scalable, community-based models that enhance access to care, continuity of services, and interprofessional collaboration in resource-limited settings.
- Embedding disaster preparedness training within mobile clinic operations may strengthen workforce readiness, interprofessional coordination and community response capacity.

**Public health implications—What are the key implications or messages for practitioners, policy makers and/or researchers in public health?**

- Sustainable reimbursement mechanisms, integrated health-system partnerships, and workforce training investments are needed to expand mobile and telehealth-enabled care models in rural communities.
- Future research should focus on longitudinal, multi-site evaluations examining patient outcomes, disaster preparedness, deployment readiness, and healthcare system impacts associated with nurse-led mobile clinic programs.

**Abstract:**

Background: Rural communities face persistent healthcare barriers related to workforce shortages, geographic isolation, transportation limitations and constrained emergency response capacity. Nurse-led mobile clinics may support healthcare access, continuity of care, and disaster preparedness in underserved settings. This study examined the Texas A&M University (TAMU) nurse-led mobile clinic model with respect to rural service delivery, health equity, operational considerations, and disaster preparedness. Methods: A mixed-methods descriptive program evaluation was conducted using programmatic operational data, survey responses, and preparedness-planning records. The TAMU mobile clinic serves six rural counties through primary, preventive, and behavioral healthcare delivery using in-person care, telehealth, and home visits. Disaster preparedness activities were integrated through the annual Disaster Day interprofessional simulation involving approximately 600–700 learners. A 2025 Central Texas flooding event served as a case study to evaluate operational preparedness and system readiness. Results: Mobile clinic operations supported healthcare access, continuity of care, and community engagement in rural settings. Interprofessional education simulation findings demonstrated perceived gains in teamwork, triage, communication, and rapid decision-making. During the 2025 flooding event, activation protocols were initiated; however, deployment was not authorized, highlighting system-level constraints related to administrative approval pathways despite operational readiness and workforce preparedness. Conclusions: Nurse-led mobile clinics may serve as an adaptable infrastructure for improving rural healthcare access, supporting continuity of care, and strengthening disaster preparedness. Findings further emphasize that clinical preparedness alone is insufficient without coordinated administrative processes, interoperable systems, and governance structures capable of supporting rapid emergency deployment.

## 1. Introduction

Access to quality healthcare remains a significant challenge in rural communities worldwide. Geographic isolation, shortages of healthcare providers, limited transportation infrastructure, and inadequate emergency-response systems contribute to persistent health disparities, resulting in higher rates of preventable morbidity and mortality in these populations [1,2,3]. These structural barriers impede timely access to primary care, preventive services, and urgent medical interventions, highlighting the need for innovative and adaptable models of care delivery to improve health equity and resilience.

Mobile clinics have emerged as a promising solution to these challenges by delivering essential healthcare services directly to underserved communities [4,5,6]. By reducing transportation barriers, improving timely treatment, and facilitating continuous access to care, mobile clinics leverage the expertise of nursing professionals to provide culturally responsive, patient-centered care while fostering trust and engagement within local communities [4,5,6]. In parallel, telehealth has demonstrated effectiveness in improving access, continuity of care, and clinical outcomes across diverse populations [7,8,9].

Beyond addressing routine healthcare needs, mobile clinics are increasingly recognized for their role in emergency preparedness and disaster response. Their mobility and adaptability enable rapid deployment to areas affected by natural disasters or public health crises, ensuring that vulnerable populations receive critical care when local infrastructure is compromised [10]. In addition, mobile clinics often engage community members in health promotion and preparedness initiatives, strengthening local capacity to respond to emergencies and contributing to broader public health resilience.

This study examines how nurse-led mobile clinics may support healthcare access, equity, and emergency preparedness in rural Central Texas. Specifically, we examine (1) service delivery and access, (2) impact on access and health equity, (3) policy and operational considerations, and (4) contributions to disaster preparedness. Through this analysis, we aim to inform policy and practice to advance rural health equity and resilience.

Disaster preparedness training in nursing education has increasingly emphasized simulation-based and competency-driven approaches to improve readiness, communication, and response capability among healthcare professionals [11,12,13,14]. Although evidence suggests that simulation-enhanced disaster training may improve perceived preparedness and foundational response competencies, important gaps remain regarding long-term implementation, operational integration, and system-level readiness in real-world emergency settings [15,16].

Rural central Texas faces a myriad of challenges, including restrictive NP scope of practice laws that limit the reach of nurse-led clinics, significant primary care workforce shortages, and inadequate emergency preparedness among health care providers. Likewise, this region is vulnerable to severe weather events such as flooding, tornadoes, and extreme heat. Updated National Competency Standards demand action and make this study timely.

We further describe operational and system-level barriers identified during the 2025 Central Texas flooding event and explore how these experiences may inform future emergency response planning and deployment readiness. By comparing preparedness simulation activities with real-world operational constraints, this study highlights the importance of coordinated governance structures, interoperable systems, and workforce readiness in supporting effective rural emergency response infrastructure.

## 2. Methods

### 2.1. Study Setting and Mobile Clinic Structure

The Texas A&M University (TAMU) nurse-led mobile clinic program operates across rural Health Professional Shortage Areas (HPSAs) (i.e., indicating provider shortages in primary care, dental health, or mental health care) in the Brazos Valley region of Texas. The weekly clinic schedule and service locations are summarized in Table 1. The program serves six counties (Houston, Washington, Leon, Grimes, Robertson, and Fayette), each characterized by limited access to primary care services. Rural residents face notable health inequalities, such as shorter life expectancies and increased rates of chronic illnesses, mental health problems, and avoidable hospital admissions. Table 1 presents the clinic operation schedules alongside the population figures for each county.

Each mobile unit is staffed by a multidisciplinary team consisting of a Family Nurse Practitioner, a Registered Nurse, and a Community Health Worker, enabling comprehensive and culturally responsive care delivery. Partnerships with local Independent School Districts (ISDs) position schools as central access points, facilitating care for school-aged children, families, and the broader community. Clinics operate on a rotating weekly schedule, dedicating one day per community to ensure consistent and predictable access to services.

The mobile clinics employ a hybrid care delivery model that integrates in-person and telehealth services [4,7,8,9]. Clinical services include acute and chronic disease management, preventive screenings, immunizations, children’s physicals, Head Start assessments, preparticipation sports evaluations, Department of Transportation (DOT) physicals, dental screenings, and sealant applications that occurred from August of 2023 through December of 2025. Home visits are conducted for homebound individuals, addressing common access barriers in rural populations. Telehealth extends care beyond the physical clinic setting by supporting follow-up visits, chronic disease monitoring, and behavioral health services, thereby enhancing continuity of care, reducing transportation barriers, and strengthening provider–patient relationships.

Through the integration of telehealth with community-based, nurse-led care, the TAMU mobile clinic program strengthens local healthcare capacity, supports early intervention and prevention, and advances health equity in rural communities. The weekly clinic schedule and service locations are summarized in Table 1.

### 2.2. Interprofessional Education (IPE) and Disaster Preparedness

#### 2.2.1. Disaster Day Simulation

Texas A&M University’s Disaster Day is among the largest student-led interprofessional disaster preparedness simulations in the United States, annually engaging approximately 600–700 learners from nursing, medicine, pharmacy, public health, dentistry, psychology, athletic training, veterinary medicine, and the Corps of Cadets. Conducted at Disaster City, a 52-acre emergency response training facility, the simulation immerses participants in mass-casualty, hazardous materials, and infrastructure-failure scenarios. Hundreds of volunteer patients, trained and applied with makeup, simulate a range of injuries and participate in these exercises. Area hospitals, emergency task force teams, health departments, and fire departments are on site to assist students. No community members are recruited to specifically participate in the scenario. These experiences are designed to develop competencies aligned with workforce preparation priorities, including effectiveness, equity, policy, and disaster readiness [11,12,13,14].

Participants operate within a structured Incident Command System (ICS), assuming roles such as Incident Commander, Medical Unit Leader, Triage Unit Leader, and Behavioral Health Operations Lead. This framework requires team-based decision-making, rapid role clarification, communication under uncertainty, and prioritization in high-stress, resource-constrained environments. The TAMU mobile clinic was integrated immediately to assist with triage and treatment of injured patients. Ten mobile clinic team members participated in the event, including drivers/medical assistants, nurse practitioners, nurses, and nursing students. Evaluation findings consistently indicate increased interprofessional awareness, improved confidence in disaster response roles, and enhanced understanding of population-level responsibilities, including shelter operations, reunification processes, continuity of care, and behavioral health surge capacity [11,12,13,14].

#### 2.2.2. Integration of Mobile Clinics into Training

In recent years, the TAMU mobile clinic has been integrated into Disaster Day, providing students with applied experience in deployable, community-embedded care delivery. Mobile clinics support rapid deployment, medication access, behavioral health stabilization, and continuity of care—functions that are particularly critical in rural and underserved settings with limited fixed healthcare infrastructure. Participation in mobile-clinic-based simulation stations was associated with improved triage decision-making, chemical incident management, interprofessional communication, and operational prioritization. Qualitative feedback further emphasized the value of faculty mentorship, high-intensity learning environments, and exposure to real-world constraints.

Structural barriers to participation, including scheduling conflicts, employment obligations, and travel limitations, were most pronounced among graduate nursing students and rural learners, reflecting broader workforce challenges in disaster response. These findings underscore the need for flexible, scalable preparedness models to ensure equitable access to training for professionals most likely to serve in rural and resource-constrained environments.

### 2.3. Disaster Response Planning and Implementation

#### 2.3.1. Activation of Disaster Response Plan

In July 2025, catastrophic flooding in the Texas Hill Country prompted activation of Texas A&M University’s disaster response plan, designed to address rapid and large-scale disruptions in healthcare access. Approval was sought to deploy both the HRSA-supported and the T.L.L. Temple Foundation-supported TAMU mobile clinics to the most severely affected counties. These clinics were acquired and are operated with extramural funding by each respective organization. The primary objectives were to stabilize access to medical and behavioral health services, reduce avoidable emergency department utilization, and maintain continuity of care in areas where local systems were overwhelmed.

Coordination with federal and foundation partners, including HRSA and the T.L.L. Temple Foundation, was prioritized to ensure compliance with funding and operational requirements. Deployment planning targeted initiation of services within 48–72 h of authorization, consistent with established disaster response timelines [10].

#### 2.3.2. Deployment Logistics, Operational Planning, and System Readiness

The mobile clinic activation workflow (Table 2) outlines the end-to-end process for disaster response, including key decision points, responsible parties, and operational outputs. This structured pathway reflects a standardized approach to rapid deployment, coordination, and service delivery in emergency contexts [17,18].

During the 2025 Central Texas flooding, local authorities requested the deployment of the mobile clinics to multiple affected counties with limited prior service coverage. Despite completion of key preparatory steps outlined in the activation workflow, including feasibility assessment, staffing, and logistical coordination, activation was not authorized by the Texas A&M Health Science Center (HSC), and deployment did not occur.

At the time of the request, a multidisciplinary response team had been assembled, including nursing providers, licensed mental health clinicians, and telehealth personnel. Operational planning included coordination with emergency management agencies, schools, and community health centers, as well as arrangements for temporary housing and site access. Telehealth infrastructure was configured to operate alongside in-person services to support continuity of care for residents affected by flooding, including those experiencing transportation barriers or geographic isolation due to infrastructure disruption. A disaster plan proposal was prepared and sent to funding institutions, outlining the scope of work, proposed service plan, timeline, communities served, financial considerations, expected impact, and accountability measures.

Planned services included triage, acute and chronic disease management, preventive screenings, immunizations, behavioral health stabilization, and wrap-around support for displaced and high-risk populations. Integration of telehealth with onsite care was intended to minimize service disruptions, maintain continuity in patient–provider relationships, reduce avoidable emergency department utilization, and support community resilience.

Although patient-level data were not collected due to non-deployment, routine operational data from the mobile clinic program support the feasibility of delivering these services in rural settings. This experience underscores the importance of pre-established administrative authorization pathways, interoperable data systems, and proactive workforce deployment strategies to ensure that prepared resources can be effectively mobilized during future emergencies.

#### 2.3.3. System Gaps and Preparedness Needs

Analysis of emergency response planning and prior activation efforts identified several system-level gaps affecting mobile clinic deployment and data capture. Operational data were primarily derived from driver logs, GPS tracking systems, and deployment records, which provided key indicators such as deployment speed, routing patterns, and patient reach.

However, limitations in documentation and data integration constrained the ability to assess service continuity and unmet need. Inconsistent billing practices and disruptions to standard workflows during emergency conditions hindered accurate estimation of service volume. Additionally, the absence of mechanisms to track individuals unable to access services limited the evaluation of access gaps and equity considerations.

Qualitative reports from staff further highlighted the challenges associated with emergency response, including operational strain and increased demand for behavioral health support among providers affected by community-level impacts. These findings underscore the need for proactive development of standardized data systems, interoperable documentation platforms, and workforce support structures to enhance preparedness and response capacity in future disaster contexts.

### 2.4. Data Collection and Analysis

Patient numbers and demographics were recorded at health fairs, school events, and clinic visits. Following their participation in the TAMU Disaster Day simulation, all nursing students were invited to scan a QR code and complete an anonymized survey about the event. No validated measurement tool was used to evaluate the performance of nursing students.

This mixed-methods descriptive program evaluation was conducted between January 2024 and July 2025 using historic programmatic operational data, routine clinical documentation, post-simulation surveys, and emergency preparedness planning records. Following participation in Disaster Day, nursing students were invited to voluntarily complete an anonymous electronic survey accessed through a QR code distributed onsite at the conclusion of the simulation event. The survey used a convenience sampling approach and was designed for program-evaluation purposes rather than hypothesis testing.

This study was guided by implementation science and community-engaged approaches to evaluate real-world healthcare delivery and disaster preparedness. The RE-AIM framework informed assessment of reach, effectiveness, and implementation across routine and emergency service contexts, while the Consolidated Framework for Implementation Research (CFIR) supported examination of organizational and contextual factors influencing deployment processes [17,18]. RE-AIM and CFIR were used as organizing frameworks to guide interpretation of implementation processes, operational reach, preparedness activities, and contextual barriers rather than as formal measurement frameworks.

Clinical service data were extracted from electronic health records (EHRs) and accompanying primary care documentation generated during mobile clinic encounters. Variables included patient demographics (age, sex, race/ethnicity, and insurance status), presenting concerns, and services delivered. Follow-up care was documented when subsequent visits occurred within the mobile clinic system. External referrals were recorded as referral events; however, due to limitations in cross-system data integration, verification of referral completion was not possible.

Interprofessional education (IPE) outcomes were assessed through both institution-wide and mobile clinic–specific processes. Institutional IPE measures, including assessments of teamwork, communication, and role clarity, were collected centrally through the Health Science Center Disaster Day simulation. Additional unit-specific measures captured nursing student competencies, perceived readiness, and discipline-specific skill development within the mobile clinic context.

Emergency response metrics were assessed using operational logs, GPS tracking systems, and deployment records. Key indicators included deployment speed, routing patterns, and patient reach. A mixed-methods approach was used to evaluate student experiences during Disaster Day, incorporating post-event surveys, structured observations, and operational logs. Surveys included Likert-scale items assessing preparedness and confidence, as well as open-ended responses capturing perceptions of learning, teamwork, and simulation effectiveness.

Open-ended survey responses and operational reflections were analyzed using conventional content analysis. Responses were independently reviewed to identify recurring concepts related to preparedness, teamwork, communication, operational barriers, and learning experiences. Codes were iteratively grouped into broader thematic categories through team discussion and consensus. Given the descriptive program-evaluation focus and small qualitative sample, formal qualitative software was not used.

### 2.5. Ethical Considerations

This study was determined to be exempt by two TAMU Institutional Review Board (IRB) reviews, STUDY 2025-0301, and 2022-1467, as it involved analysis of programmatic, operational, and de-identified data not constituting human subjects research. All clinical services were delivered under standard care protocols, with informed consent procedures embedded within routine clinical workflows. Patient data were stored within secure, encrypted electronic health record systems or maintained in protected mobile clinic records in accordance with institutional policies and federal privacy standards.

## 3. Results

Results are presented across three distinct but related domains: (1) routine mobile clinic operations involving patient service delivery across the six-county region, (2) interprofessional education and disaster preparedness outcomes among Disaster Day student participants, and (3) operational disaster-response readiness during the 2025 Central Texas flooding event.

### 3.1. Routine Mobile Clinic Operations

Routine operations of the TAMU nurse-led mobile clinic demonstrated broad reach and comprehensive service delivery across the six-county region. The clinic served a demographically diverse patient population, varying in age, insurance status, and socioeconomic background, reflecting the heterogeneous needs of rural communities.

Services included acute and chronic disease management, preventive screenings, immunizations, children’s physicals, Department of Transportation (DOT) physicals, and dental health services. Telehealth utilization supported follow-up care, chronic disease monitoring, and behavioral health consultations, serving as a critical mechanism for maintaining continuity of care between in-person clinic rotations. This hybrid model reduced gaps in service delivery, mitigated transportation barriers, and supported sustained provider–patient relationships in resource-limited settings.

Table 3 summarizes demographic and clinical characteristics of patients receiving services through routine mobile clinic operations and does not represent Disaster Day student participants.

### 3.2. Interprofessional Education (IPE) Outcomes

#### 3.2.1. Disaster Preparedness and Competency Development

Student responses following participation in the Disaster Day simulation reflected generally positive perceptions of preparedness and confidence. The 76.2% of participants reported feeling better prepared or somewhat better prepared, and 61.9% reported more confident or somewhat more confident in their ability to respond to disaster scenarios. However, 23.8% of respondents reported neutral perceptions, indicating that they felt neither more prepared nor less prepared following the experience (Table 4).

Triage was consistently (45.5%) identified as the most salient skill acquired, with students emphasizing rapid patient categorization (e.g., red, yellow, green, black) as a key learning outcome. Interprofessional learning emerged as a significant strength of the simulation, with 70% of the respondents answering strongly or somewhat agree. Participants further reinforced improved teamwork and collaboration across disciplines, including learning from more advanced trainees and recognizing the importance of coordinated, team-based care in disaster settings.

Participants frequently identified interprofessional communication and triage decision-making as meaningful learning outcomes. One participant noted that the experience improved their understanding of “teamwork with various healthcare professionals,” while another emphasized learning how to rapidly prioritize patients during mass-casualty scenarios. Responses were anonymous; therefore, participant identifiers were not assigned.

#### 3.2.2. Learning Environment and Structural Barriers

Despite these strengths, several barriers to engagement were identified. Students reported limitations related to the number and diversity of patient scenarios, perceived disorganization, unclear instructions, and insufficient supplies. These factors contributed to intermittent periods of inactivity, reducing both the realism of the simulation and its perceived educational value.

Participants recommended increasing the number of simulated patients, improving logistical coordination, and enhancing communication across participating disciplines. These findings indicate that while the simulation effectively supports skill development in triage and teamwork, improvements in operational structure and scenario complexity may further enhance student engagement and learning outcomes.

#### 3.2.3. Mobile Clinic Specific Learning Outcomes

Integration of the mobile clinic into Disaster Day produced distinct learning benefits. Students assigned to the mobile clinic reported perceived gains in triage decision-making, chemical incident management, and interdisciplinary communication compared to experiences in other simulation stations.

Qualitative feedback emphasized the value of high-intensity learning environments, faculty mentorship, and exposure to real-world constraints associated with rural disaster response. Structural barriers to participation, including scheduling conflicts, work obligations, and travel constraints, were most pronounced among nursing students and rural learners, reflecting broader workforce challenges in emergency preparedness.

Table 5 presents examples of student responses. Evidence was evaluated according to the specific competency question. For instance, if a student answered what they considered the most valuable lesson, it was taken as a sign of positive progress, whereas any mention of challenges or obstacles pointed to areas needing improvement.

### 3.3. Emergency Response During the 2025 Flooding Event

#### 3.3.1. Deployment Readiness and Administrative Constraints

The 2025 flooding event revealed important gaps in the mobile clinic’s capacity for rapid deployment. While the program maintained operational readiness and established partnerships with local agencies, administrative pathways required for activation at the institutional level were insufficient to support timely deployment.

Other university-affiliated units successfully mobilized and coordinated with local jurisdictions. In contrast, despite preparedness at the clinical and operational levels, the mobile clinic was not authorized for activation. This limitation highlights the critical role of institutional decision-making structures in determining the effectiveness of disaster response.

#### 3.3.2. Coordination and System-Level Barriers

Although interprofessional disaster preparedness training addressed both natural and human-made hazards, administrative constraints limited real-world implementation. Existing partnerships across Central Texas positioned providers to respond, and telehealth systems were capable of rapid deployment. However, institutional barriers restricted the mobile clinic’s ability to transition from preparedness to active service delivery.

Similar constraints affected other university-affiliated groups, limiting participation to outreach and support roles rather than direct clinical care. These findings underscore the importance of aligning operational readiness with administrative and policy-level decision-making structures.

#### 3.3.3. System Gaps and Implications for Emergency Response

Although the mobile clinic did not deploy during the flooding event, the experience generated critical insights into system-level gaps affecting emergency response. Existing operational data systems—including driver logs, GPS tracking, and deployment records—captured key indicators such as deployment speed, routing patterns, and patient reach.

However, limitations in data integration and documentation workflows restricted the ability to assess service continuity and unmet need. Inconsistent billing practices and disruptions during emergency conditions impeded the accurate estimation of service delivery. Additionally, the absence of mechanisms to track individuals unable to access care limited the assessment of equity and population-level impact.

Qualitative reports from staff further highlighted workforce-related challenges, including operational strain and increased demand for behavioral health support among providers affected by community-level impacts. These findings emphasize the need for proactive development of standardized data systems, interoperable documentation platforms, and workforce support structures to strengthen future emergency response capacity.

## 4. Discussion

### 4.1. Rural Health Access and Equity

This study highlights the persistent structural barriers faced by rural communities in accessing timely and equitable healthcare, including geographic isolation, workforce shortages, and gaps in preventive and primary care services [1,2,3]. The Texas A&M University (TAMU) nurse-led mobile clinic model directly addresses these challenges by delivering routine, preventive, and chronic care services within underserved communities. The program’s consistent weekly presence across designated counties, combined with telehealth and home-visit capabilities, reduces transportation barriers and supports continuity of care—two critical determinants of rural health equity [7,8,9].

By integrating care delivery within trusted community settings, such as schools, and partnering with local school districts, the model expands access for children, families, and working adults, enhancing both social and geographic accessibility. The use of nurses and community health workers further promotes trust and cultural alignment, which is particularly important in communities with limited engagement with traditional healthcare systems [4,5,6]. In disaster contexts, the capacity to deploy services to unaffected or underserved areas offers an additional equity benefit by maintaining access for populations whose local systems are disrupted [10]. Collectively, these findings support the role of nurse-led mobile clinics as mechanisms to reduce access gaps, promote equity, and strengthen community resilience.

### 4.2. Effectiveness of Mobile Clinics in Routine and Emergency Contexts

The TAMU mobile clinic program demonstrates a dual role in enhancing care delivery and system responsiveness. In routine settings, the clinics provide comprehensive primary care services, including chronic disease management, acute care, preventive screenings, immunizations, children’s physicals, and dental services. The integration of telehealth extends care beyond scheduled clinic rotations, supporting follow-up care, chronic disease monitoring, and behavioral health services. This hybrid model contributes to continuity of care and helps address structural barriers common in rural settings [7,8,9].

Program data further illustrate the clinic’s reach and service profile. Since Fall 2023, the clinics have delivered 1348 in-person visits and 23 telehealth consultations. Telemedicine is an emerging complementary approach to providing patient care when a physical clinic is not available within a community. Preventive services, including immunizations and sports physicals, were among the most frequently requested services, reflecting community demand for accessible, school-based and community-based care. Chronic conditions commonly addressed included hypertension, diabetes, hyperlipidemia, and anxiety, while acute care visits frequently involved sinusitis and seasonal allergies. Preventive screenings, including cervical and breast cancer services, were also provided.

However, findings also highlight ongoing challenges. Depression screening was conducted for 157 patients, yet no patients met criteria for remission within one year, underscoring persistent gaps in behavioral health management and continuity of care in resource-limited settings. These findings likely reflect broader continuity of care, follow-up, referral access, and documentation challenges common in rural behavioral health management. Additionally, limitations are noted due to incomplete demographic reporting—particularly related to gender identity, sexual orientation, and income—limited the ability to fully assess disparities and population-level impact.

Beyond clinical care, the mobile clinic contributes to community capacity through targeted education and training initiatives. In response to identified needs, the program delivered Stop the Bleed training to 131 participants and hygiene education to 120 elementary school students. These activities extend the impact of the clinic beyond direct care, supporting prevention and community preparedness.

In emergency contexts, mobile clinics offer significant potential as adaptable healthcare delivery platforms. Their ability to provide triage, chronic disease management, behavioral health support, and telehealth-enabled continuity of care positions them as valuable assets during disruptions to local healthcare infrastructure [10]. However, as demonstrated during the 2025 Central Texas flooding event, operational readiness alone is insufficient without corresponding administrative authorization and system-level coordination. These findings emphasize that successful emergency response depends not only on clinical capacity but also on institutional alignment and decision-making structures.

### 4.3. Interprofessional Education and Workforce Preparedness

The integration of mobile clinics into Disaster Day provides a valuable model for interprofessional education (IPE) in disaster preparedness. Simulation-based training environments have been shown to improve disaster readiness, teamwork, and response confidence among healthcare trainees [11,12,13,14]. Consistent with prior research, students in this study reported increased confidence and improved interprofessional collaboration following participation in the simulation.

Mobile-clinic-based training environments offer distinct advantages by exposing learners to real-world constraints, including limited resources, time pressure, and complex coordination requirements. These experiences support the development of adaptive problem-solving skills that are essential for rural and disaster response contexts.

At the same time, participation in IPE activities revealed structural barriers, including scheduling conflicts, employment obligations, and travel constraints, particularly among nursing students and rural learners. These barriers reflect broader workforce challenges and highlight the need for more flexible and accessible training models. Strengthening interprofessional training infrastructure and reducing barriers to participation will be critical to preparing a workforce capable of responding effectively in resource-constrained and emergency settings.

### 4.4. System Readiness and Implementation Considerations

Findings from the 2025 flooding event demonstrated that operational and clinical preparedness alone are insufficient to ensure successful disaster deployment. While the mobile clinic program demonstrated workforce readiness, logistical coordination capacity, and telehealth capability, institutional authorization barriers prevented transition from preparedness to active service delivery. This disconnect highlights the need for alignment between clinical operations and institutional governance structures.

An important finding from this evaluation was the distinction between clinical preparedness and systemic deployment capacity. Although the mobile clinic program demonstrated workforce readiness, interprofessional coordination, telehealth capability, and operational planning capacity, institutional authorization barriers limited real-world deployment during the 2025 flooding event. These findings suggest that effective disaster preparedness requires not only trained personnel and mobile infrastructure, but also clearly defined administrative pathways, interoperable communication systems, and coordinated governance structures capable of supporting rapid activation during emergencies.

Data system limitations further constrained the evaluation of service delivery and unmet need. Existing documentation systems captured key operational metrics but lacked integration across systems, limiting the ability to track continuity of care, referral outcomes, and populations unable to access services. These gaps are particularly important in disaster contexts, where timely and accurate data are essential for decision-making and resource allocation.

Workforce considerations also emerged as a critical component of system readiness. Staff reported increased operational strain and emotional burden during disaster response conditions, particularly when affected by the same community-level impacts as patients. These findings emphasize the importance of incorporating workforce support strategies, including mental health resources and staffing flexibility, into emergency preparedness planning.

Collectively, these findings position mobile clinics as dual-use infrastructure capable of supporting both routine care delivery and emergency response. However, realizing this potential requires coordinated investment in administrative processes, interoperable data systems, and workforce support mechanisms. These considerations align with broader implementation science frameworks emphasizing the role of context, system readiness, and organizational alignment in determining successful program adoption and sustainability [17,18].

### 4.5. Limitations

This study should be interpreted considering several limitations. First, the findings are based on an observational, programmatic evaluation of a single mobile clinic model, which may limit generalizability to other settings, populations, or healthcare systems. While the Texas A&M University mobile clinic program operates across multiple rural counties, variations in infrastructure, workforce capacity, and community context may influence the transferability of these findings.

Second, data collection relied in part on self-reported survey responses and qualitative feedback, particularly in the evaluation of the Disaster Day simulation. Although these methods provide valuable insight into participant perceptions and experiential learning, they are subject to response bias and may not fully capture objective changes in competency or performance. Because survey participation was voluntary and anonymous, response rates could not be linked to individual learner characteristics or longitudinal outcomes. The absence of validated measurement tools and standardized assessment frameworks further limits the ability to quantify learning outcomes and compare findings across settings. Because this study was observational and programmatic in nature, findings should be interpreted as descriptive and exploratory rather than causal demonstrations of intervention effectiveness. Accordingly, findings related to disaster response should be interpreted as indicators of preparedness and implementation capacity rather than causal evidence of emergency response effectiveness.

Third, incomplete demographic data, including non-disclosure of gender identity, sexual orientation, and income, restricted the ability to fully characterize the populations served and assess disparities in access or outcomes. While respecting patient autonomy and privacy is essential, these gaps highlight challenges in balancing trust with the need for comprehensive data to inform equity-focused analyses. In addition, subsequent follow-up after external referral cases was not possible, causing an absence of longitudinal or comparative data.

Fourth, the relatively small proportion of telehealth encounters compared to in-person visits limits conclusions regarding the effectiveness and scalability of telehealth within this hybrid model. Additional research is needed to better understand the role of telehealth in extending care continuity in rural and resource-constrained environments.

Finally, although the inclusion of a real-world disaster case study strengthens the practical relevance of this work, the inability to deploy mobile clinic services during the 2025 Central Texas flooding limits conclusions regarding operational effectiveness in active emergency response. Instead, findings from this event primarily reflect organizational readiness and system-level constraints rather than direct clinical impact.

Additionally, because mobile clinic deployment did not occur during the 2025 Central Texas flooding event, findings related to disaster response primarily reflect operational preparedness, planning capacity, and system-level constraints rather than direct measures of deployed clinical effectiveness. Despite these limitations, this study offers important insights into the role of nurse-led mobile clinics as integrated platforms for care delivery, workforce development, and disaster preparedness. Future research incorporating longitudinal designs, objective performance measures, and multi-site comparisons will be critical to strengthening the evidence base and informing scalable, sustainable models of mobile healthcare.

### 4.6. Implications for Practice and Policy and Mobile Healthcare Sustainability

Findings from this study highlight the need to strengthen operational, educational, and policy frameworks to support mobile healthcare delivery in rural and disaster-prone settings. In practice, nurse-led mobile clinics demonstrate the value of a structured hybrid model that integrates in-person services with telehealth to improve access and continuity of care [7,8,9]. Expansion of this approach may reduce gaps in service delivery between clinic rotations, particularly for patients managing chronic conditions or facing transportation and access barriers. Standardized workflows for preventive screening, chronic disease management, follow-up care, and telehealth integration are essential to ensure consistent, high-quality service delivery across diverse communities. Locating mobile clinics within trusted community settings, including schools and local health agencies, can enhance accessibility, strengthen community trust, and support proactive engagement that mitigates healthcare disruptions during emergencies [4,5,6]. To improve preparedness, mobile clinic programs should establish proactive deployment protocols with predefined service areas, transportation logistics, and communication strategies to support rapid and coordinated response during crises [10].

Education and workforce development are central to sustaining mobile healthcare models. Integrating mobile clinics into immersive, interprofessional training environments, including high-fidelity simulation, provides learners with applied exposure to disaster response and rural healthcare delivery. These experiences support the development of key competencies such as triage, prioritization, systems thinking, and interprofessional communication [11,12,13,14]. Addressing structural barriers, including scheduling constraints, travel requirements, and competing work obligations, is necessary to ensure equitable participation, particularly among nursing and rural learners who are often essential to frontline response capacity. Leveraging mobile clinics as experiential training platforms can strengthen workforce readiness to deliver flexible, community-centered, and equity-oriented care in complex and resource-constrained environments.

Sustained policy support is essential to ensure the long-term viability and effectiveness of mobile healthcare programs. Lessons from the 2025 Central Texas flooding underscore the importance of clearly defined administrative pathways for rapid deployment, including authorization processes and coordination mechanisms with funding partners and emergency management systems. Sustainable financing models should incorporate federal and state funding streams, rural health infrastructure investments, and public–private partnerships to support operations, telehealth capacity, workforce development, and disaster preparedness. Reimbursement policies must adequately account for mobile and telehealth services to maintain financial sustainability in low-volume rural settings [7,8,9]. In addition, robust documentation infrastructure—including interoperable electronic health records and data-sharing agreements—is necessary to support continuity of care, referral tracking, and evaluation across routine and emergency contexts. These system-level considerations align with implementation science frameworks emphasizing the importance of context, infrastructure, and organizational readiness in determining program sustainability and scalability [17,18].

Anticipating region-specific vulnerabilities, such as transportation disruptions, telecommunications limitations, and behavioral health service gaps, further enables mobile clinics to align services with community needs and strengthen system resilience. Overall, the sustainability and impact of mobile healthcare programs depend on coordinated advancements across practice, education, and policy domains. Expanding hybrid care delivery models, strengthening interprofessional workforce training, improving administrative readiness for disaster response, and aligning policy and financing structures with rural healthcare realities will position mobile clinics as integral components of equitable, resilient, and community-centered healthcare systems.

## 5. Conclusions

The Texas A&M University (TAMU) nurse-led mobile clinic model represents an adaptable approach to addressing persistent gaps in rural healthcare access, equity, and emergency preparedness. Through routine operations across underserved counties, the program demonstrates the capacity to reduce geographic and transportation barriers, enhance continuity of care through hybrid in-person and telehealth delivery, and support culturally responsive engagement through nurses and community health workers [4,5,6,7,8,9]. These findings reinforce the role of mobile clinics as scalable models for extending primary care and preventive services in resource-limited settings.

Integration of mobile clinics into interprofessional disaster preparedness training further highlights their value as dual-purpose infrastructure supporting both care delivery and workforce development. High-fidelity simulation environments, such as Disaster Day, provide realistic contexts for developing competencies in triage, communication, leadership, and population-level decision-making—skills essential for effective disaster response [11,12,13,14]. Expanding the use of mobile clinics within training programs may strengthen workforce preparedness, particularly for providers serving rural and disaster-prone communities.

The 2025 Central Texas flooding event underscored both the potential and limitations of mobile clinic deployment in real-world emergencies. Although clinical activation did not occur due to administrative constraints, the experience revealed critical system-level gaps related to authorization pathways, coordination processes, and data infrastructure. These findings emphasize that clinical readiness alone is insufficient; effective emergency response requires alignment across operational, administrative, and policy domains [10,17,18].

Future research should extend beyond cross-sectional and simulation-based evaluations to examine longitudinal outcomes across the full disaster management cycle, including preparedness, response, recovery, and mitigation. There is a need for objective performance measures to assess competency development and operational effectiveness, as well as for consistent integration and evaluation of national and international competency frameworks [15,16]. Establishing evidence-based performance standards and embedding them within educational and accreditation systems may strengthen disaster preparedness across the nursing workforce.

Findings from this descriptive program evaluation suggest that nurse-led mobile clinics may support rural healthcare access, interprofessional preparedness training, and operational disaster-response readiness. However, the 2025 flooding event highlighted that clinical preparedness alone is insufficient without coordinated institutional authorization pathways and deployment infrastructure capable of supporting rapid emergency activation. Future disaster planning frameworks should distinguish between workforce and clinical preparedness versus institutional deployment authority and governance readiness.

Collectively, this study demonstrates that nurse-led mobile clinics can advance rural health equity, support community resilience, and provide a flexible platform for integrated care delivery and disaster response. Sustaining and scaling these models will require continued investment in hybrid care delivery, interprofessional training, administrative readiness, and supportive policy frameworks. By addressing these interconnected domains, mobile clinics can function as essential components of resilient, equitable healthcare systems capable of responding effectively across all phases of emergency preparedness.

## Figures and Tables

**Table 1:** Brazos Valley Mobile Clinic Weekly Schedule by County and Community.

Day	County (Population)	Texas Community	Time	Notes
Monday	Washington (37,007)	Brenham	9 a.m.–3 p.m.	
Tuesday	Leon (16,538)	Jewitt	9 a.m.–3 p.m.	
Wednesday	Washington/Fayette (37,007/36,474)	Burton/Round Top-Carmine	9 a.m.–3 p.m.	Alternates weekly
Thursday	Grimes (32,384)	Iola	9 a.m.–3 p.m.	
Friday	Robertson (17,267)	Hearne	9 a.m.–3 p.m.	

Mobile Clinic Services Include: Primary medical care for adults and children Acute and chronic disease management Laboratory testing Immunizations (e.g., influenza, routine childhood, and adult vaccines) Women’s health services, including screenings and Health education and referral services Point-of-care testing (e.g., COVID-19, influenza, streptococcal infection).

**Table 2:** Mobile Clinic Activation & Disaster Response Workflow.

Phase	Trigger/Decision Point	Key Actions	Responsible Party	Outputs
1. Situation Awareness	Event notification or request for support (e.g., flood, wildfire, outbreak)	Receive alerts from partners; assess location, impacted population, access constraints, and immediate needs	Program manager/mobile clinic leadership	Situation report (SITREP); request summary
2. Feasibility and Scope	Determination of whether deployment is safe, feasible, and timely	Conduct rapid needs assessment; identify site; define service package (e.g., triage, acute/chronic care, behavioral health, telehealth)	Clinical and operations leads	Proposed mission scope; draft staffing and supply plan
3. Authorization	Decision to activate and reallocate mobile clinic resources	Submit request through institutional leadership; confirm compliance, legal, and safety requirements	Institutional leadership	Go/no-go decision; activation record
4. Mobilization	Activation approved; initiate deployment (typically within 48–72 h)	Assign staff roles; prepare vehicles, supplies, and equipment; ensure communication and documentation systems; coordinate logistics	Operations lead	Staffing plan; supply manifest; route and site plan
5. Onsite Operations	Arrival and initiation of service delivery	Establish clinical workflow; deliver services; coordinate with emergency response systems; track metrics and referrals; provide telehealth support	Clinical lead with local coordination partners	Daily SITREP; encounter records; referral log
6. Demobilization	Decision to conclude or transition operations	Coordinate care handoff; restock supplies; conduct equipment and vehicle maintenance; support staff debrief and recovery	Clinical and operations leads	Demobilization checklist; restocking report
7. After-Action and Improvement	Post-deployment or post-decision review	Conduct debrief; analyze performance and barriers; update protocols, training, and partnerships	Program leadership and stakeholders	After-action report; improvement plan

**Table 3:** Participant Demographic and Clinical Characteristics of Patients.

Variable	Category	Percentage (%)
Age (years)	<27 years	39
	27–54 years	29
	54–82 years	31
	>82 years	1
Sex Assigned at Birth	Male	18
	Female	25
	Unknown	57
Insurance Type	Commercial	47
	Medicaid	13
	Medicare	13
	Self-pay	26
	Other	1
Top Diagnoses	Hypertension	31
	Immunization	24
	Hyperlipidemia	11
	Sports physical exam	11
	Anxiety	5

Note: Percentages may not total 100% due to rounding or missing data.

**Table 4:** Qualitative Themes Identified From Student Responses Regarding Key Learning Outcomes.

Qualitative Theme Identified	Supporting Participant Responses	Frequency (n)	Percentage
Triage and patient categorization identified as a primary learning outcome	Responses referencing triage, prioritization, or red/yellow/green/black tagging	5 of 11	45.5%
Interprofessional teamwork and collaboration identified as a meaningful aspect of learning	Responses referencing interdisciplinary teamwork, collaboration with other healthcare professionals, or learning from peers	4 of 11	36.4%
Communication identified as an important learning outcome	Responses specifically referencing communication or talking with healthcare professionals	2 of 11	18.2%
PPE and disaster safety awareness identified as a key learning outcome	Responses specifically referencing PPE or disaster safety procedures	1 of 11	9.1%
Efficiency and prioritization under pressure identified as important	Responses referencing efficiency, prioritization, or rapid decision-making	2 of 11	18.2%

Percentages are based on respondents who provided substantive open-ended qualitative responses regarding the most important thing learned during Disaster Day 2025 (n = 11). Some responses reflected more than one theme; therefore, percentages may total greater than 100%.

**Table 5:** Interprofessional Education Competency Outcomes Following Disaster Day Simulation.

IPE Competency	Evidence of Gain	Example Student Response
Teamwork	Positive	“Teamwork with various healthcare professionals”
Communication	Positive	“Communication”
Preparedness	Mixed	“I am more confident in my ability to respond…”
Collaboration	Positive	“Learning with older nursing students improved my experience”
Barriers/Obstacles	Noted	“Disorganization”, “Unequal team dynamics”

## Data Availability

The datasets presented in this article are not readily available because of Personal Health Information (PHI) and contractual agreements at community sites. Further inquiries can be directed to the corresponding author.
